# Comparing record linkage software programs and algorithms using real-world data

**DOI:** 10.1371/journal.pone.0221459

**Published:** 2019-09-24

**Authors:** Alan F. Karr, Matthew T. Taylor, Suzanne L. West, Soko Setoguchi, Tzuyung D. Kou, Tobias Gerhard, Daniel B. Horton

**Affiliations:** 1 RTI International, Research Triangle Park, NC, United States of America; 2 Center for Pharmacoepidemiology and Treatment Science, Institute for Health, Health Care Policy and Aging Research, Rutgers University, New Brunswick, NJ, United States of America; 3 Sidney Kimmel Medical College, Thomas Jefferson University, Philadelphia, PA, United States of America; 4 Bristol-Myers Squibb, Hopewell, NJ, United States of America; Victoria University, AUSTRALIA

## Abstract

Linkage of medical databases, including insurer claims and electronic health records (EHRs), is increasingly common. However, few studies have investigated the behavior and output of linkage software. To determine how linkage quality is affected by different algorithms, blocking variables, methods for string matching and weight determination, and decision rules, we compared the performance of 4 nonproprietary linkage software packages linking patient identifiers from noninteroperable inpatient and outpatient EHRs. We linked datasets using first and last name, gender, and date of birth (DOB). We evaluated DOB and year of birth (YOB) as blocking variables and used exact and inexact matching methods. We compared the weights assigned to record pairs and evaluated how matching weights corresponded to a gold standard, medical record number. Deduplicated datasets contained 69,523 inpatient and 176,154 outpatient records, respectively. Linkage runs blocking on DOB produced weights ranging in number from 8 for exact matching to 64,273 for inexact matching. Linkage runs blocking on YOB produced 8 to 916,806 weights. Exact matching matched record pairs with identical test characteristics (sensitivity 90.48%, specificity 99.78%) for the highest ranked group, but algorithms differentially prioritized certain variables. Inexact matching behaved more variably, leading to dramatic differences in sensitivity (range 0.04–93.36%) and positive predictive value (PPV) (range 86.67–97.35%), even for the most highly ranked record pairs. Blocking on DOB led to higher PPV of highly ranked record pairs. An ensemble approach based on averaging scaled matching weights led to modestly improved accuracy. In summary, we found few differences in the rankings of record pairs with the highest matching weights across 4 linkage packages. Performance was more consistent for exact string matching than for inexact string matching. Most methods and software packages performed similarly when comparing matching accuracy with the gold standard. In some settings, an ensemble matching approach may outperform individual linkage algorithms.

## Introduction

Linkage among medical databases such as electronic health records (EHRs), health insurer claims, and patient-generated data is becoming increasingly important for delivering high-quality, high-value healthcare; conducting valid and generalizable research; and evaluating healthcare policy. In countries with fragmented healthcare systems, such as the United States, linkage of EHRs across multiple healthcare settings and institutions enables clinicians to access information arising from care provided in other systems, which can improve continuity and efficiency of care and reduce redundancy.

Additionally, linkage among different kinds of data—such as EHR, registries, and claims—can provide clinicians and researchers with access to complementary sources of information. For example, EHR-derived data on prescribed drugs, vital signs, laboratory results, and smoking and alcohol histories combined with claims-based data on dispensed drugs and out-of-system diagnoses and encounters, can provide a more comprehensive picture of a patient’s care than information from either dataset alone.

Consequently, database linkage can help create comprehensive, longitudinal datasets with information on patients’ conditions and treatments over time. In addition to their utility in clinical care, such approaches can be applied to research, allowing investigators to access richer datasets and in the process overcome selection, information, and confounding biases [1–4]. Also, multiple databases can be linked by personal identifiers such as name, address, and date of birth. These variables are subject to several forms of error, and they may change over time, including address, last names upon marriage, and evolving classifications of race and gender. Whereas use of national or universal identifiers, such as Social Security numbers, facilitates more direct linkage between databases, access to these sensitive identifiers is often restricted and may still be imperfect because of errors in data entry, process, and transfer. Linkage errors resulting from inappropriate matching of different individuals or incomplete matching can lead to bias [5–7].

Conceptually, all record linkage algorithms operate similarly. First, a set of *linking variables* is designated that is common to both datasets, which provides a basis for comparing individual records from each dataset. Second, a numerical weight is calculated for each compared pair (one record from each database), which is interpreted as the degree of confidence that paired records represent the same person or entity. Finally, a matching threshold is calculated or specified, and pairs whose weights exceed the designated threshold are declared to be matches.

Typically, linkage variables are either numeric or text, such as string variables, where matching of these variables is done using exact or inexact methods. Exact string matching requires that two strings match exactly, character-by-character, including capitalization and any other characters such as hyphens, accents, or spaces. By contrast, inexact string matching, which has multiple versions, assigns a numerical similarity based on criteria such as the number of insertions, deletions, and replacements needed to convert one string to the other.

Methods for converting string comparisons to weights may be classified as either *deterministic* or *probabilistic* [8]. In the most basic version of deterministic record linkage, the weight for a pair is simply the number of linking variables with exact agreement, yielding values ranging from 0 to the number of linking variables. Probabilistic weight determination uses statistical modeling based on estimated probabilities that records match, given equality or similarity of linking variables [9]. The M-probability for a given linking variable is the probability of exact agreement on that variable given two matching records—a true positive match. The U-probability is the probability of agreement given that the records do not match—a false positive match. Probabilities of a match typically depend on the frequency of linking variables values, either within the dataset or in external benchmark datasets. For instance, gender or common surnames are more likely to match by chance—resulting in higher U-probabilities—than unusual surnames. Determination of M- and U- probabilities may be specified exogenously, reflecting past experience or expert opinion (e.g., the Fellegi-Sunter approach [9]) or calculated endogenously (e.g., using the expectation-maximization [EM] algorithm [10]).

Numerous record linkage programs exist, which differ with respect to cost and methodologic transparency (open-source as compared with proprietary), operating system/hardware requirements, and scalability. Conceptually, all linkage programs perform string comparison, weight determination, and match determination. Data preprocessing is a key step in record linkage, including purging of duplicate records, harmonization of linkage variables (which is necessary, for instance, if the common values of gender are “F” and “M” in one, but “1” and “2” in the other), and common representation of missing values. Blocking is a common strategy to reduce computational burden, where only pairs of records that agree on one or more *blocking variables* are compared. If the blocking variable has *n* values, both time and memory requirements are reduced by a factor of *n*.

Most studies on linkage performance use only one software package to link synthetic or real-world databases [11–15]. Although these studies provide valuable information on linkage challenges, accuracy, and biases, they do not account for all the complexities of the linkage process or the variability across different linkage packages and approaches. Little has been published on the comparative behavior and output of software programs that can be used to link healthcare databases. One study used actual identifiers to evaluate probabilistic approaches from two software packages (Link Plus and Link King) without studying how specific variables affected weights and matches [16]. Another study created synthetic datasets to compare the quality and performance of 10 different linkage packages but did not examine the impact of different matching thresholds [17]. Neither study examined the performance of algorithms within and across software packages. Consequently, the present study aimed to compare 17 linkage methods within 4 nonproprietary available linkage programs to determine how the quality of dataset linkage is affected by linkage algorithm, blocking variable selection, methods for string matching and weight determination, and decision rules for pair matching.

## Materials and methods

We compared the performance of 4 linkage software packages applied to real patient data from university-affiliated institutions. We focused on variables typically available in real healthcare data (e.g., name, gender, date of birth (DOB)) that contain actual errors but with very low levels of missingness (see also S1 File). The Rutgers University Institutional Review Board deemed this project not to be human subjects research as defined by 45 CFR 46. Nonetheless, we implemented strict security measures to preserve patient privacy and confidentiality in accordance with institutional regulatory and legal requirements. Results were de-identified before being shared with investigators outside of Rutgers University (see Analysis files). No health-related information was used for this study, and no patient identifiers were viewed by investigators or others not employed by Rutgers University.

### Datasets

We used data for the three years 2013–2015 contained in noninteroperable EHRs from two neighboring, clinically affiliated but administratively separate institutions. The inpatient dataset (IPD) came from the Robert Wood Johnson University Hospital, a 965-bed urban teaching hospital with approximately 30,000 admissions per year. As received, the IPD included demographic data on all patients admitted overnight to the hospital during the study period. Each hospital admission resulted in a distinct entry; consequently, individuals with repeated hospitalizations had multiple entries. The outpatient dataset (OPD) came from the Rutgers Robert Wood Johnson Medical School, which has a multispecialty outpatient medical practice of over 500 affiliated physicians. The OPD contained information about all patients seen at least once during the study period, with only one record per person, based on a unique outpatient medical record number (MRN), representing the most recent set of demographic data. Both the IPD and OPD included first name, last name, DOB, gender, race, street address, city, state, and ZIP Code. Because only the OPD contained information on ethnicity, we excluded this variable from linkage experiments. Because the datasets were from clinically affiliated institutions, administrators used a proprietary linkage package to assign common MRNs. An inpatient MRN accessible within the OPD was used as a gold standard to evaluate linkage accuracy.

We preprocessed both datasets to harmonize the variable names and values. Preprocessing entailed dropping variables not used in the linkage runs or other analyses (e.g., street address, date of visit), reclassifying race (e.g., Asian, black, white, other, or missing), and extracting year of birth (YOB) from DOB. We converted implausible values—such as ZIP Codes containing letters—to missing values, but we did not standardize names.

After data preprocessing, we proceeded with deduplication. The OPD contained 176,154 records of purportedly unique individuals, making deduplication unnecessary. We deduplicated the original 104,289 IPD records by removing entries that matched exactly on 6 variables: MRN, last name, first name, gender, YOB, race, and ZIP Code. Records containing a missing ZIP Code were retained only if no other record matching on all other identifiers had a valid ZIP Code. The final IPD and OPD datasets contained the following variables: last name, first name, gender, DOB, YOB, age, race, ZIP Code, and MRN. We also assigned a unique study identifier to each record.

### Software packages

We selected software packages based on multiple criteria: (1) available for a Windows-based computer, (2) nonproprietary, (3) described in prior publications, (4) containing reasonable documentation with some transparency regarding default settings, (5) capable of operating in scripting/batch mode, and (6) capable of saving weights for compared pairs. Based on these criteria, we chose 4 software packages: R (Version 3.4.0, *RecordLinkage* package), Merge ToolBox (MTB, Version 0.75), Curtin University Probabilistic Linkage Engine (CUPLE, shortened in figures and tables to CU), and Link Plus (LP, Version 2.0) (Table A in S1 File).

### Experiment design

Because both DOB and YOB are highly reliable variables in healthcare, we conducted 2 linkage experiments, one using DOB as the blocking variable (presented as primary analyses) and the other using YOB as the blocking variable (presented as secondary analyses). First name, last name, and gender comprised matching variables for all linkage runs. Aside from the software package, we varied linkage runs by the string matching method (exact or inexact); for inexact string matching, we applied the most common method, Jaro-Winkler. We also varied the weight determination method, using 3 probabilistic approaches (Fellegi-Sunter, expectation-maximization, EpiLink [18]), as well as deterministic linkage. Most of the software packages implement more than one weight determination method. For probabilistic linkage approaches other than expectation-maximization, we used default values of M- and U-probabilities, which for each linkage variable were typically 0.95 and the reciprocal of the number of unique values, respectively. Some packages required manual entry of these values.

### Analysis files

We prepared 2 de-identified analysis files, one for each linkage experiment, with each file containing one row of information for each compared record pair. Analysis files included columns for IPD and OPD record identifiers, gender, age (upper limit 90), and race; a variable indicating whether the record pair matched on inpatient MRN; and 17 sets of weights corresponding to each linkage run. We assembled the analysis files using R (Version 3.4.0) and SAS (Version 9.4).

To compare results across runs, we scaled the 17 sets of weights to range from 0 to 1, corresponding to the lowest and highest weights respectively. The scaling was linear and was done using the following equation:

$$ScaledWeight=\frac{OriginalWeight‐min(OriginalWeight)}{max(OriginalWeight)‐min(OriginalWeight)}$$

Additionally, we ranked the weights within runs from highest (ranked as 1) to lowest. Declaring matches based on weight rank, such as rank 1 or rank 2, also allowed for comparability across algorithms.

Using this analysis file, we investigated the 17 sets of weights and scaled weights from multiple perspectives. We conducted descriptive analyses of the weights, including display of their empirical cumulative distribution functions. We also evaluated relationships among the weights, including their correlation, principal components analysis, and accuracy with respect to the gold standard, inpatient MRN. We also compared the performance of using matching weights as decision rules, including the area under the receiver operating characteristic (ROC) curve (AUC).

## Results

The deduplicated datasets contained 69,523 inpatient records and 176,154 outpatient records, respectively. The total number of possible record pair comparisons, without blocking, was 12,199,192,962 pairs. Blocking on DOB reduced the number of record pair comparisons to 400,490. Datasets were similar based on gender distribution but distinctly different based on age and race (Table 1).

**Table 1:** Demographic characteristics by dataset.

	Inpatient (N = 69,523)	Outpatient (N = 176,154)
Age, N (%)		
<1	8,185 (11.8%)	6,127 (3.5%)
1‒9	3,123 (4.5%)	21,747 (12.3%)
10‒19	3,502 (5.0%)	24,105 (13.7%)
20‒29	6,004 (8.6%)	17,197 (9.8%)
30‒39	6,900 (9.9%)	19,992 (11.3%)
40‒49	6,058 (8.7%)	20,306 (11.5%)
50‒59	8,968 (12.9%)	24,825 (14.1%)
60‒69	9,861 (14.2%)	21,505 (12.2%)
70‒79	8,412 (12.1%)	13,063 (7.4%)
80‒89	6,654 (9.6%)	6,241 (3.5%)
90+	1,856 (2.7%)	1,046 (0.6%)
Gender, N (%)		
Female	36,753 (52.9%)	100,238 (56.9%)
Male	32,770 (47.1%)	75,911 (43.1%)
NA	0	5 (0.003%)
Race, N (%)		
Asian	6,533 (9.4%)	15,397 (8.7%)
Black	9,506 (13.7%)	22,607 (12.8%)
Other	14,242 (20.5%)	1,149 (0.7%)
White	38,619 (55.5%)	95,372 (54.2%)
NA	623 (0.9%)	41,629 (23.6%)
First name, unique values	13,221	27,232
Last name, unique values	31,103	60,014
Inpatient medical record number, unique values	69,091	138,156
Inpatient medical record number, missing values	0	37,975

### Characteristics of the weights

Table 2 summarizes the statistics for the weights arising from the 17 linkage runs, displaying the number of unique weights produced, the maximum and minimum weights, the number of pairs that received the highest and second highest weights, and the number of pairs that received the lowest and second lowest weights. As expected, exact string matching approaches generally produced fewer distinct weights (range 4–9) than inexact string matching (range 8–64,273).

**Table 2:** Summary of weights produced by record linkage using DOB as the blocking variable.

Linkage Run Name	String Matching	Weight Determination	Number of Weights	Minimum Weight	Maximum Weight	Pairs with Highest Weight	Pairs with Second Highest Weight	Pairs with Lowest Weight	Pairs with Second Lowest Weight
R/EX/FS	Exact	Prob-FS	8	-12.3808	32.35027	30,536	24	176,066	189,273
R/EX/EM	Exact	Prob-EM	8	-17.7754	25.82314	30,536	24	176,066	189,273
R/EX/EPI	Exact	Prob-EPI	8	0	1	30,536	24	176,066	189,273
MTB/EX/FS	Exact	Prob-FS	9	-12.3808	32.35027	30,536	24	176,056	10
MTB/EX/EM	Exact	Prob-EM	9	-17.7681	25.82333	30,536	24	176,056	10
MTB/EX/D	Exact	Det	4	0	3	30,536	4,445	176,066	189,443
CU/EX/FS	Exact	Prob-FS	9	-12.3808	32.35027	30,536	24	176,048	10
LP/EX/FS	Exact	Prob-FS	9	-7.53877	12.78385	30,536	24	176,056	10
LP/EX/EM	Exact	Prob-EM	9	-7.53877	12.78385	30,536	24	176,056	10
R/INEX/FS	Inexact	Prob-FS	8	-12.3808	32.35027	31,619	25	176,018	189,095
R/INEX/EM	Inexact	Prob-EM	121	-18.5957	22.80771	30,536	3	176,018	189,095
MTB/INEX/FS	Inexact	Prob-FS	64,273	-12.3808	32.35027	30,536	3	5,691	1
MTB/INEX/EM	Inexact	Prob-EM	64,273	-17.7789	22.76115	30,536	3	5,691	1
MTB/INEX/D	Inexact	Det	24,603	0	3	30,536	3	5,692	1
CU/INEX/FS	Inexact	Prob-FS	1,492	-12.3808	32.35027	30,554	3	173,105	2
LP/INEX/FS	Inexact	Prob-FS	37	2.1	15.8	15	3	^a^	^a^
LP/INEX/EM	Inexact	Prob-EM	37	2.1	15.8	15	3	^a^	^a^

DOB, date of birth; R, R package; MTB, Merge ToolBox; CU, Curtin University Probabilistic Linkage Engine; LP, Link Plus; Prob-FS, probabilistic, Fellegi-Sunter; Prob-EM, probabilistic, expectation-maximization; Prob-EPI, probabilistic, EpiLink; Det, deterministic.

^a^ We were unable to recover negative weights for Link Plus with inexact string matching.

The empirical cumulative distribution functions of scaled weights varied considerably across the 17 linkage runs, confirming that these methods behaved differently (Fig A in S1 File).

### Relationships among weights

Although there was substantial agreement among the 9 algorithms that use exact string matching, they did not produce identical rankings (Table 3; Table B in S1 File). All runs with exact string matching assigned the highest weight to the same 30,536 pairs that matched on first name, last name, gender, and DOB. Probabilistic string-matching algorithms besides expectation-maximization (i.e., Fellegi-Sunter and EpiLink) assigned higher weight to pairs that matched on last name and gender than pairs matching on first name and gender (Table 3). Software packages differed subtly in how they handled missing data (here, gender).

**Table 3:** Agreement on matching variables for runs with exact string matching, blocking on DOB.

Weight Rank	Agreement on					
	R/EX/FS	R/EX/EM	R/EX/EPI	MTB/EX/FS	MTB/EX/EM	CU/EX/FS
1	First, Last, Gender	First, Last, Gender	First, Last, Gender	First, Last, Gender	First, Last, Gender	First, Last, Gender
2	First, Last	First, Last	First, Last	First, Last	First, Last	First, Last
3	Last, Gender	First, Gender	Last, Gender	Last, Gender	First, Gender	Last, Gender
4	First, Gender	Last, Gender	First, Gender	First, Gender	Last, Gender	First, Gender
5	Last	First	Last	Last	First	Last
6	First	Last	First	First	Last	First
7	Gender	Gender	Gender	Gender	Gender	Gender
8	None	None	None	None, gender missing	None, gender missing	None, gender missing
9	N/A	N/A	N/A	None, no matching variables missing	None, no matching variables missing	None, no matching variables missing

DOB, date of birth; R, R package; FS, probabilistic, Fellegi-Sunter; EM, probabilistic, expectation-maximization; EPI, probabilistic, EpiLink; MTB, Merge ToolBox; CU, Curtin University Probabilistic Linkage Engine

**Fig 1:**
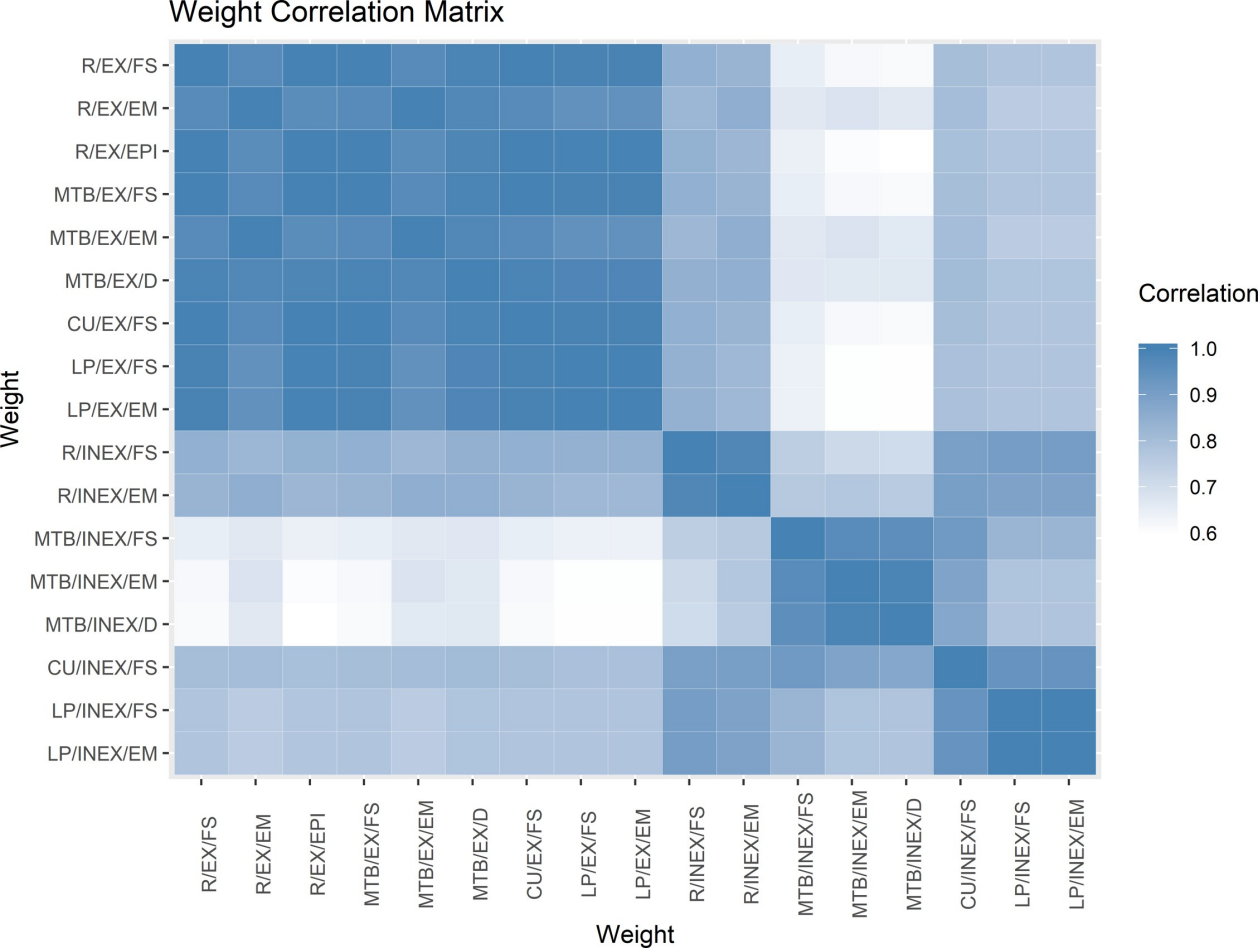
Correlation matrix for the 17 sets of weights. EX, exact string matching; INEX, inexact string matching; R, R package; MTB, Merge ToolBox; CU, Curtin University Probabilistic Linkage Engine; LP, Link Plus; FS, probabilistic, Fellegi-Sunter; EM, probabilistic, expectation-maximization; EPI, probabilistic, EpiLink; D, deterministic. The rows and columns are ordered so that runs using exact methods are at the top and left.

All runs using exact methods were highly correlated (Fig 1). Among runs using inexact methods, only those run in CUPLE and R were highly correlated with the exact methods. Runs using inexact string matching in the other two software packages (LP and MTB) were correlated with other runs using the same software but much less so with runs in other packages. The principal components analysis indicated only four predominant dimensions to the 17 sets of weights, whereby the first four principal components explained 98.97% of the variation among the weights (Table C in S1 File).

### Comparison with medical record number check

Among 30,536 record pairs matching on DOB, first name, last name, and gender across most runs, 809 pairs (representing 1.16% of IPD, 0.45% of OPD) did not match on MRN (Table 4). Manual review of a random sample of 86 these 809 pairs suggested that these likely were the same individual with either different MRNs (77%), a missing MRN in OPD (22%), or a misspecified MRN in OPD (1%). Consequently, the gold standard itself had a small error rate of approximately 0.5% to 1%.

**Table 4:** Agreement with gold standard among records with the highest weights, blocking on DOB.

Linkage Run Name	String Matching	Weight Determination	Number (%) of Pairs with Highest Weight		Number (%) of Pairs with First or Second Highest Weight	
			Agreement with inpatient MRN			
			No	Yes	No	Yes
R/EX/FS	Exact	Prob-FS	809 (2.6)	29,727 (97.4)	814 (2.7)	29,746 (97.3)
R/EX/EM	Exact	Prob-EM	809 (2.6)	29,727 (97.4)	814 (2.7)	29,746 (97.3)
R/EX/EPI	Exact	Prob-EPI	809 (2.6)	29,727 (97.4)	814 (2.7)	29,746 (97.3)
MTB/EX/FS	Exact	Prob-FS	809 (2.6)	29,727 (97.4)	814 (2.7)	29,746 (97.3)
MTB/EX/EM	Exact	Prob-EM	809 (2.6)	29,727 (97.4)	814 (2.7)	29,746 (97.3)
MTB/EX/D	Exact	Det	809 (2.6)	29,727 (97.4)	2,597 (7.4)	32,384 (92.6)
CU/EX/FS	Exact	Prob-FS	809 (2.6)	29,727 (97.4)	814 (2.7)	29,746 (97.3)
LP/EX/FS	Exact	Prob-FS	809 (2.6)	29,727 (97.4)	814 (2.7)	29,746 (97.3)
LP/EX/EM	Exact	Prob-EM	809 (2.6)	29,727 (97.4)	814 (2.7)	29,746 (97.3)
R/INEX/FS	Inexact	Prob-FS	945 (3.0)	30,674 (97.0)	951 (3.0)	30,693 (97.0)
R/INEX/EM	Inexact	Prob-EM	809 (2.6)	29,727 (97.4)	809 (2.6)	29,730 (97.4)
MTB/INEX/FS	Inexact	Prob-FS	809 (2.6)	29,727 (97.4)	809 (2.6)	29,730 (97.4)
MTB/INEX/EM	Inexact	Prob-EM	809 (2.6)	29,727 (97.4)	809 (2.6)	29,730 (97.4)
MTB/INEX/D	Inexact	Det	809 (2.6)	29,727 (97.4)	809 (2.6)	29,730 (97.4)
CU/INEX/FS	Inexact	Prob-FS	816 (2.7)	29,738 (97.3)	816 (2.7)	29,741 (97.3)
LP/INEX/FS	Inexact	Prob-FS	2 (13.3)	13 (86.7)	3 (16.7)	15 (83.3)
LP/INEX/EM	Inexact	Prob-EM	2 (13.3)	13 (86.7)	3 (16.7)	15 (83.3)

DOB, date of birth; MRN, medical record number; R, R package; MTB, Merge ToolBox; CU, Curtin University Probabilistic Linkage Engine; LP, Link Plus; Prob-FS, probabilistic, Fellegi-Sunter; Prob-EM, probabilistic, expectation-maximization; Prob-EPI, probabilistic, EpiLink; Det, deterministic.

We also noted a small number of record pairs with very low weights despite matching MRNs, representing either different people with the same MRN or errors in the matching variables (Table 5). Record pairs that agreed only on DOB and MRN but not on first name, last name, or gender occurred only for 1 record pair in 1 software package using inexact string matching. Approximately 400 to 500 record pairs (about 1.2% of pairs with the second lowest weights across multiple runs) matched only on DOB and gender but not on first or last name. Manual review of a random sample (n = 100) of these records suggested that 99% were likely the same individuals who did not match appropriately. In most pairs (80%), this occurred with newborns that had a first name of “Male” or “Female” only in IPD and differed on last names, presumably the mothers’ in IPD and fathers’ or compound last names in OPD. Other discrepancies occurred because of various issues with names, most often misspellings in first names and inconsistent representation of compound last names.

**Table 5:** Agreement with gold standard among records with the lowest weights, blocking on DOB.

Linkage Run Name	String Matching	Weight Determination	Number (%) Pairs with Lowest Weight		Number (%) Pairs with First or Second Lowest Weight	
			Agreement with inpatient MRN			
			No	Yes	No	Yes
R/EX/FS	Exact	Prob-FS	176,066 (100)	0 (0)	364,871 (99.9)	468 (0.1%)
R/EX/EM	Exact	Prob-EM	176,066 (100)	0 (0)	364,871 (99.9)	468 (0.1%)
R/EX/EPI	Exact	Prob-EPI	176,066 (100)	0 (0)	364,871 (99.9)	468 (0.1%)
MTB/EX/FS	Exact	Prob-FS	176,056 (100)	0 (0)	176,066 (100)	0 (0)
MTB/EX/EM	Exact	Prob-EM	176,056 (100)	0 (0)	176,066	0 (0)
MTB/EX/D	Exact	Det	176,066 (100)	0 (0)	365,038	471 (0.1%)
CU/EX/FS	Exact	Prob-FS	176,048 (100)	0 (0)	176,058	0 (0)
LP/EX/FS	Exact	Prob-FS	176,056 (100)	0 (0)	176,066	0 (0)
LP/EX/EM	Exact	Prob-EM	176,056 (100)	0 (0)	176,066	0 (0)
R/INEX/FS	Inexact	Prob-FS	176,018 (100)	0 (0)	364,693	420 (0.1%)
R/INEX/EM	Inexact	Prob-EM	176,018 (100)	0 (0)	364,693	420 (0.1%)
MTB/INEX/FS	Inexact	Prob-FS	5,691 (100)	0 (0)	5,692	0 (0)
MTB/INEX/EM	Inexact	Prob-EM	5,691 (100)	0 (0)	5,692	0 (0)
MTB/INEX/D	Inexact	Det	5,692 (100)	0 (0)	5,693	0 (0)
CU/INEX/FS	Inexact	Prob-FS	173,105 (100)	0 (0)	173,107	0 (0)
LP/INEX/FS	Inexact	Prob-FS	84 (98.8)	1 (1.2)	^a^	^a^
LP/INEX/EM	Inexact	Prob-EM	84 (98.8)	1 (1.2)	^a^	^a^

DOB, date of birth; MRN, medical record number; R, R package; MTB, Merge ToolBox; CU, Curtin University Probabilistic Linkage Engine; LP, Link Plus; Prob-FS, probabilistic, Fellegi-Sunter; Prob-EM, probabilistic, expectation-maximization; Prob-EPI, probabilistic, EpiLink; Det, deterministic.

^a^ Low weights were unrecoverable in Link Plus using inexact string matching.

### Comparative performance of the methods

**Fig 2:**
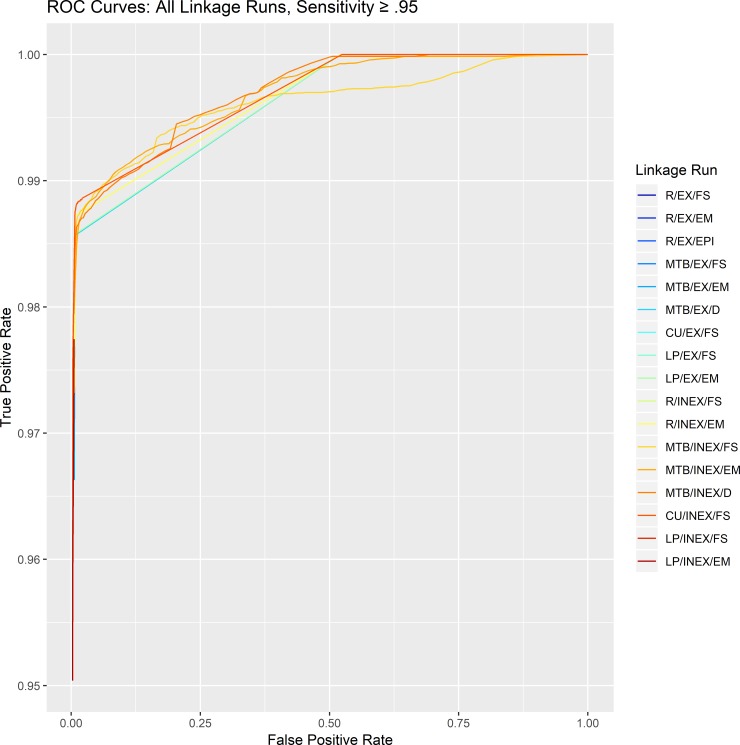
Receiver operating characteristic curves for all linkage runs. EX, exact string matching; INEX, inexact string matching; R, R package; MTB, Merge ToolBox; CU, Curtin University Probabilistic Linkage Engine; LP, Link Plus; LP, Link Plus; FS, probabilistic, Fellegi-Sunter; EM, probabilistic, expectation-maximization; EPI, probabilistic, EpiLink; D, deterministic. Curves are limited to values of sensitivity equal to or exceeding 0.95 for clarify. Full ROC curves are presented in Fig D in S1 File.

We compared the performance of the packages and algorithms using scaled weights and the gold standard, inpatient MRN, to identify declared matches, false positive matches, and false negative matches as the weight threshold varied. Across of range of scaled weights, the number of declared matches of record pairs varied among different packages and algorithms (Fig B and C in S1 File). When declared matches were the record pairs with the highest weights, most linkage algorithms performed similarly well, with sensitivity > 90%, specificity > 99%, positive predictive value (PPV) > 97%, and negative predictive value (NPV) > 99% (Table D in S1 File). Expansion of declared matches to include those with the second highest weights did not substantively change the test characteristics for most runs (Table E in S1 File). The ROC curves reflected these high levels of accuracy, with AUC greater than 0.99 for most linkage runs and minor differences among them (Fig 2; Fig D and Table F in S1 File).

## Ensemble methods

We also explored whether the 17 linkage runs could be combined into an “ensemble” method that outperformed all runs individually. The motivation came from ensemble methods in machine learning, such as Super Learner [19], in which multiple models of the data are constructed and decisions are made by combining the results from these models. We explored two plausible approaches to ensemble methods based on: (1) averaging scaled weights and (2) “vote counting” using Rank 1 or 2 weights (i.e., pairs assigned the highest or second highest weights).

### Average scaled weight

As the name connotes, the average scaled weight is the average of the 17 individual weights:

$$AverageScaledWeight=\frac{1}{17}\left(Weight\_R13\_Scaled+\ldots +Weight\_LP13\_Scaled\right)$$

As measured by AUC, the average scaled weight ensemble method outperformed all individual linkage runs (0.9962 vs. ≤ 0.9948) (Fig E in S1 File).

### Rank 1 or 2 voting

An alternative ensemble method was based on the number of linkage runs that assigned the highest or second highest weight to a record pair (see S1 File: Ensemble method: Rank 1 or 2 voting). The AUC for this ensemble method was 0.9807, lower than with average scaled weight and lower than some individual linkage runs (Fig E in S1 File).

## Year of birth experiment

In recognition that DOB is not available in all databases or accurate in all circumstances, we performed additional experiments blocking on YOB (S1 File: Year of birth experiment). All 9 exact string matching linkage runs assigned the highest weight to the same 30,805 pairs, as compared with 30,536 pairs when blocking on DOB (Table G in S1 File). These additional 269 pairs matched on first name, last name, gender, and YOB, but not day of birth, month of birth, or both. Using YOB instead of DOB as the blocking variable, we gained 43 matches that shared a common MRN but also added 226 matches that did not match on MRN (Table H in S1 File). Correspondingly, blocking on YOB led to marginal improvements in sensitivity and NPV at the expense of specificity and PPV (Table I in S1 File). Additionally, blocking on YOB imposed major computational challenges because of the 329-fold increase in the number of compared pairs (131,906,591). Compared to the efficient DOB linkage runs that took less than one minute (Table J in S1 File), some YOB linkage runs took more than one hour (Table K in S1 File). Additionally, we were unable to complete any runs using R with inexact string matching due to computation limitations. Thus, compared to the experiment blocking on DOB, blocking on YOB seemed ineffective, yielding 5 times more additional errors than correct matches while substantially increasing computational time and precluding certain analyses.

## Discussion

Using real data from noninteroperable EHRs, we performed a comprehensive assessment of the behavior and usability of nonproprietary available linkage software, evaluating the decision-making capability of specific linkage methods such as type of string comparison and weight determination and output from the linkage runs. Across multiple runs, we found relatively few perceptible differences in matching results, specifically with respect to ranks of the highest weights. Performance among software packages using exact string matching varied much less than that for methods using inexact string matching. From other perspectives, such as declaring matches to be pairs assigned the highest weight, linkage runs with inexact string matching were notably less efficient. As seen in Table 2, the linkage runs with exact string matching identified the same number of highest weight records, whereas some linkage runs with inexact string matching produced either too many matches or, in the case of LP, dramatically too few—possibly because only positive weights could be included in output. An ensemble matching approach had incrementally superior accuracy to individual algorithms.

The performance of most software packages and algorithms was similar, although not identical, with respect to matching accuracy as compared with our gold standard. In our linkage runs, exact matching using EM algorithms for weight determination appeared to be slightly less reliable than other exact matching algorithms: EM algorithms prioritized matching on first name over matching on last name, a more diverse and specific matching variable. Why some linkage runs prioritized first name over last name or vice versa is unclear. Compared with exact matching, linkage runs of inexact matching algorithms led to more variability in both the number of discrete weights assigned (8 to 64,273) and the number of record pairs receiving the highest weight (15 to 31,619). Our linkage runs also revealed more variability in assigning low weights than high weights when using both exact string matching and inexact string matching. This diversity among low-weight record pairs is unlikely to affect declared matches at common matching thresholds; however, it does underscore the differences in weight determination among approaches.

We focused on the weights associated with the record pairs evaluated for each linkage program, evaluating linkage programs and algorithms as decision tools rather than the actual matching decisions. The findings suggest that the selection of weight thresholds for declaring matches can have a substantial impact on both operational and inferential uses of the linked data [20, 21]. Choosing a threshold also depends on the study objectives. If false-positive linkages are costly, whether monetarily, scientifically, or in terms of human health, then higher matching thresholds may be preferred at the cost of lower sensitivity. For other questions, such as estimating the prevalence of rare diseases, even a few false negatives may cripple an analysis and the resulting loss of statistical power may be prohibitive [5, 11–15, 22, 23].

A prior study similarly compared the performance and accuracy of different linkage approaches at different matching thresholds [16]. However, that study was limited to only three approaches (2 probabilistic and 1 deterministic) without mention of specific probabilistic matching algorithms or the impact of individual matching variables on weight determination. Another more extensive comparison of the performance of both open-source and proprietary linkage packages used synthetic datasets to develop a method for evaluating data linkage software [17]. After comparing both computational speed and linkage quality of various software packages, that study identified a couple of packages, which were not named, that outperformed others. However, unlike our current findings, the prior work did not directly evaluate the performance of specific algorithms or the impact of specific variables or weight thresholds on decision-making.

Even small numeric differences in weighting could be more important in some settings, such as work with low-quality, incorrect data, or high levels of missingness. Prior work has shown that probabilistic approaches, which are often more time consuming, generally perform better in settings with low-quality data or high levels of missingness [8]. Although the matching variables in our datasets contained few missing values, treatment of missing values varied among the 4 software packages used in the present study. Furthermore, some packages did not allow the user to change default settings regarding missing data; other packages even lacked documentation about the subject. These differences in the handling of missing data may have important implications for the performance and reliability of linkage approaches, a hypothesis that bears further investigation.

Unlike prior work [17], we did not formally assess the computational performance of the packages. For the main experiment blocking on DOB, all ran within 1 minute (Table J in S1 File). For the experiment that blocked on YOB, only MTB and CUPLE ran smoothly for choices of string matching and weight determination (Table K in S1 File). LP ran relatively efficiently, in part because only positive weights were calculated for inexact string matching. R did not run at all for inexact string matching or for exact string matching with EpiLink weight determination, presumably because R is single-threaded and holds all objects in (real or virtual) memory. This experience highlights the importance of suitable blocking variables.

The extent to which our findings can be generalized to other datasets is uncertain. Like all real datasets, the two datasets we used had some data quality problems. As one such indicator, nearly 3% (Table 4) of the highest ranked matches did not share the same MRN, our gold standard. Other demographic variables in the original datasets that were not used as linking variables had high levels of missingness, such as ethnicity and address. Nonetheless, in terms of the linking variables tested, both datasets seemed to be rather good quality, in part perhaps because of human health and financial incentives [24]. Furthermore, evaluating linkage error using a gold standard—even an alloyed gold standard—provides important information on linkage quality [7].

Another factor that may limit the generalizability of the findings is that we chose not to do extensive data cleaning beyond deduplication [21, 25, 26]. Specifically, we did not perform name standardization—such as removing name suffixes like “Jr,” conducting nickname lookups, and dealing with compound surnames and name transpositions—because we felt it was beyond the scope of the current project. Based on our manual review of low-weight record pairs with the same MRN, name standardization would likely have improved results for many of the linkage packages. Nonetheless, such processes may come at a cost, as heavy cleaning may decrease overall linkage quality [25]. However, we found one other group with low matching weights despite agreements in the gold standard: infants whose first name and last name changed between the inpatient and outpatient settings. This specific group underscores the importance of understanding real-world healthcare practices—such as the naming of newborn infants in inpatient settings as “Male” or “Female” with mothers’ surnames—when interpreting EHR data [27]. Other analogous circumstances such as name changes with marriage or divorce may also compromise matching accuracy based on names. It is important to note that these findings may not be generalizable to other populations, such as the elderly.

We also did not explore the effect of using additional linking variables such as address because of the challenges in standardizing address text and concerns about the reliability of address variables, which can and do change over time. Identifying and accounting for identifier errors when linking data, especially on ZIP Code, helps to reduce bias caused by linkage errors [28]. Further, we did not examine the effects of varying numerical parameters—such as values of M-probabilities and U-probabilities—or thoroughly investigate how different choices of linking variables would affect linkage quality. Some of the results suggest that, in the same way as weight thresholds matter less than weight rank, some algorithms may be relatively insensitive to the choice, for instance, of M-probabilities and U-probabilities. Conducting weight-focused experiments similar to the current study could help resolve these kinds of questions.

## Conclusion

We assessed the behavior and performance of various linkage algorithms using nonproprietary available linkage software and real data from two EHR systems. In settings in which levels of missing data are low and data quality is high, exact string matching approaches vary little across software packages, although approaches using exogenous weight determination, such as Fellegi-Sunter, may outperform those with endogenous methods, such as EM algorithms. With few exceptions, most linkage runs with either exact string matching or inexact string matching yielded similar groups of higher-weighted record pairs with high accuracy. Where possible, blocking on DOB seems preferable to blocking on YOB, given its greater computational efficiency and greater accuracy. Certain ensemble methods appear to improve overall performance of the algorithms.

## Supporting information

S1 File Supporting tables, figures and text. (PDF)
