# Supplementary material for: Comparing record linkage software programs and algorithms using real-world data
Source: PLoS One. 2019 Sep 24;14(9):e0221459. doi: 10.1371/journal.pone.0221459 (PMC6759179; doi:10.1371/journal.pone.0221459)
Supplement: S1 File — (PDF) [file pone.0221459.s001.pdf]

Supporting Information for:

“Comparing record linkage software programs and algorithms using real-world data”

Karr AF et al.

Additional details about materials and methods

Because of the large numbers of record pairs being compared and the computer processing and memory required for linkage, we chose datasets between 100,000 to 200,000 records apiece. Additionally, we used datasets that we knew to have an ample number of records in common. We used a 64-bit Windows 10 operating system on a Dell Optiplex 7050 having an Intel® Core™ i7-7700 CPU with 4 cores running at 3.60 GHz, 64GB of RAM, and a solid-state drive.

Table A. Linkage software packages evaluated

Package	Version	Requires	Origin	Download URL
R package<br>Core System<br>R<br>RecordLinkage	 3.4.0<br>3.4.0<br>0.4-10	R	The open source R RecordLinkage package from R Studio[1]	https://mirrors.nics.utk.edu/cran/<br>https://mirrors.nics.utk.edu/cran/<br>https://cran.r-project.org/web/packages/RecordLinkage/index.html
Merge ToolBox	0.75	Java	German Record Linkage Center	http://soz-159.uni-duisburg.de/software/index.html
Curtin University Probabilistic Linkage Engine	-	Windows Powershell	LinXmart, Curtin University Population Linkage Engine	Obtained directly from Curtin University
Link Plus	2.0		Centers for Disease Control and Prevention [2]	ftp://ftp.cdc.gov/pub/Software/RegistryPlus/Link_Plus/RPLinkPLus-2.0.exe

Empirical cumulative distribution functions

We produced empirical cumulative distribution functions (ECDFs) of scaled weights for the 17 linkage runs (Fig A). For each value of scaled weight (x), the corresponding y-value is the fraction of pairs with scaled weight less than or equal to x. The number of weights is always finite; consequently, all of these are step functions. However, when the number of weights is large, as for some of the inexact string matching methods, the ECDFs appear to be continous. More rapid increases in the ECDF (i.e., closer to the left of the graph) correspond to higher proportions of pairs being assigned low weights, which means that even for low thresholds, most pairs would be declared nonmatches. For a given threshold *x*, the proportion of declared *matches* is 1.0 minus the value of the ECDF at *x*. We observed this behavior for most packages and runs, where most nonmatches are determined at low thresholds. By contrast, using inexact string matching for Link Plus (LP)—and to a lesser extent, for Merge ToolBox (MTB)—the rise in the ECDF occurs only for larger values of x. Specifically, LP/INEX/FS and LP/INEX/EM yield small numbers of declared nonmatches for thresholds below 0.75.

Fig A. Empirical cumulative distribution functions of the scaled weights for the 17 linkage runs.

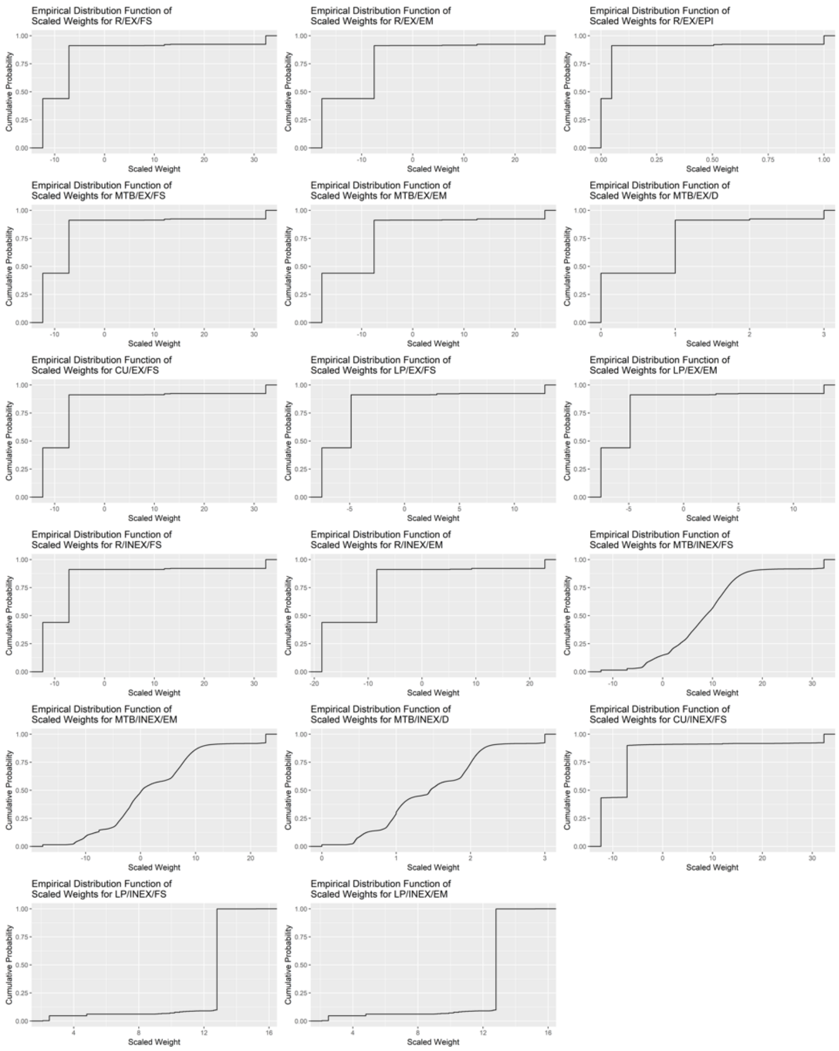

EX, exact string matching; JW, Jaro-Winkler (inexact string matching); R, R package; MTB, Merge ToolBox; C, Curtin University Probabilistic Linkage Engine; LP, Link Plus.

Relationships among weights

Among the 9 linkage runs that used exact string matching, the same record pairs tended to be grouped together by weight similarly across runs. All runs agreed on the pairs with the highest rank and, except for the run with deterministic linkage, second highest rank. However, the order and distribution of assigned weights differed by algorithm (Table B).

Table B. Concordance of pair rankings among the exact string matching methods.

Number of Pairs	Rank								
	R/EX/FS	R/EX/EM	R/EX/EPI	MTB/EX/FS	MTB/EX/EM	MTB/EX/D	CU/EX/FS	LP/EX/FS	LP/EX/EM
30,536	1	1	1	1	1	1	1	1	1
24	2	2	2	2	2	2	2	2	2
1,055	3	4	3	3	4	2	3	3	3
3,366	4	3	4	4	3	2	4	4	4
163	5	6	5	5	6	3	5	5	5
7	6	5	6	6	5	3	6	6	6
3	7	7	7	7	7	3		7	7
189,270	7	7	7	7	7	3	7	7	7
10	8	8	8	8	8	4	8	8	8
8	8	8	8	9	9	4		9	9
176,048	8	8	8	9	9	4	9	9	9

EX, exact string matching; R, R package; MTB, Merge ToolBox; CU, Curtin University Probabilistic Linkage Engine; LP, Link Plus; FS, probabilistic, Fellegi-Sunter; EM, probabilistic, expectation-maximization; EPI, probabilistic, EpiLink; D, deterministic.

Principal components analysis

The characteristics of the first 4 principal components of the 17 sets of weights are shown in Table C. Based on the loading matrix (not included here), we interpret the four principal components (PCs) as follows:

- PC1 is the high common correlation among the 17 methods;
- PC2 differentiates methods using exact string matching from methods using inexact string matching;
- PC3 picks out R/INEX/FS, R/INEX/EM, LP/INEX/FS and LP/INEX/EM among the methods using inexact string matching: those with relatively few weights;
- PC4 separates R and MTB from CU and LP.

Table C. Characteristics of the first four principal components of the 17 sets of weights.

	PC1	PC2	PC3	PC4
Standard deviation	3.787	1.325	0.721	0.456
Proportion of Variance	0.8435	0.1033	0.0306	0.0123
Cumulative Proportion	0.8435	0.9468	0.9774	0.9897

PC, principal component.

Comparative performance of the methods

We compared the numbers of declared matches of record pairs at different threshold values of scaled weights (Figs B, C). As the weight threshold increased to 0.667, MTB/EX/D was the first run to declare as matches the 30,536 record pairs matching on date of birth (DOB), first name, last name, and gender. Four other linkage runs (R/EX/FS, R/EX/EM, LP/INEX/FS, and LP/INEX/EM) reached this core set of record pairs at thresholds less than 0.8. MTB/INEX/FS, MTB/EX/D, and MTB/INEX/D required thresholds exceeding 0.995, and R/INEX/EM required a threshold of 1.0 to arrive at 30,536 matches. R/INEX/FS and CU/INEX/FS never produced 30,536 matches for any threshold value.

Fig B. Declared matches using scaled weights as a function of threshold between 0.75 and 1.

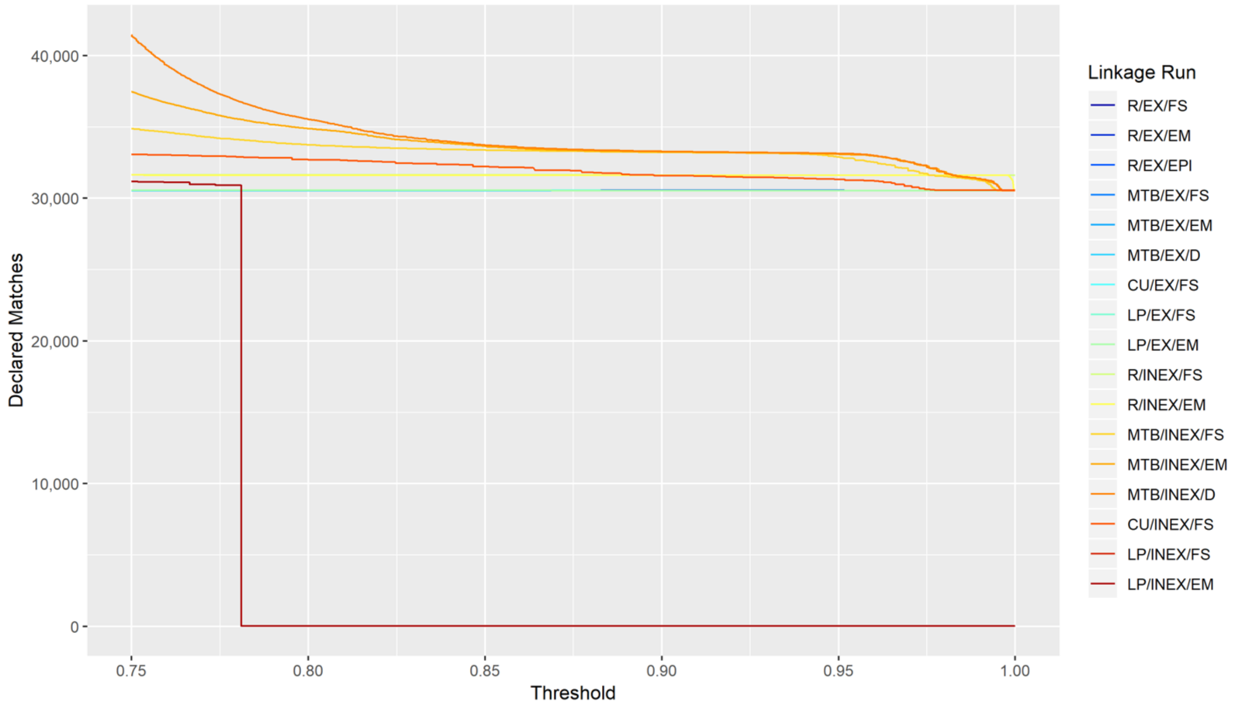

R, R package; MTB, Merge ToolBox; CU, Curtin University Probabilistic Linkage Engine; LP, Link Plus; EX, exact string matching; INEX, inexact string matching; FS, probabilistic, Fellegi-Sunter; EM, probabilistic, expectation-maximization; EPI, probabilistic, EpiLink; D, deterministic.

Fig C is an expanded version of Fig B. It shows dramatic variation among the methods for some values of the threshold.

Fig C. Declared matches using scaled weights as a function of threshold, for all thresholds.

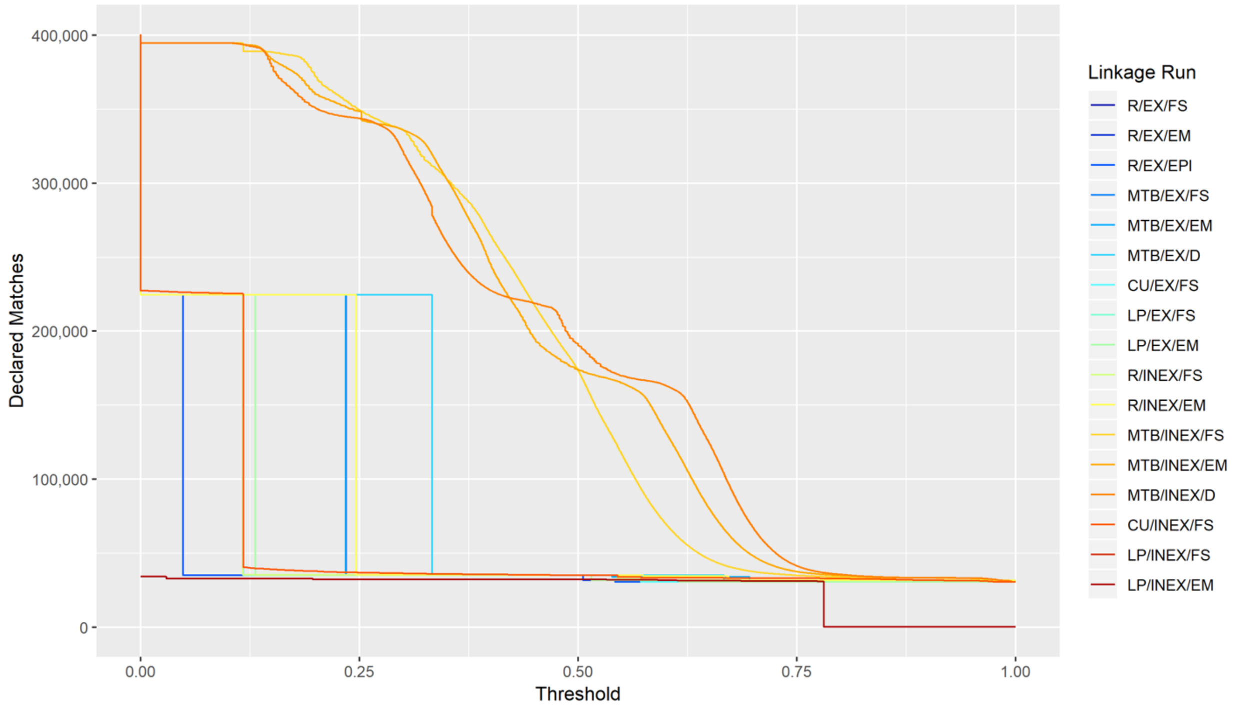

R, R package; MTB, Merge ToolBox; CU, Curtin University Probabilistic Linkage Engine; LP, Link Plus; EX, exact string matching; INEX, inexact string matching; FS, probabilistic, Fellegi-Sunter; EM, probabilistic, expectation-maximization; EPI, probabilistic, EpiLink; D, deterministic.

Tables D and E contain the standard decision rule characteristics for all 17 runs blocking on DOB, using inpatient MRN as the gold standard to establish accuracy. Table D presents the declared matches as pairs with the highest weight, whereas Table E presents the declared matches as pairs with the highest or second highest weight.

Table D. Standard decision rule characteristics for the 17 DOB linkage runs when declared matched are those with the highest weight.

Linkage Run Name	String Matching	Weight Determination	Sensitivity[a] (%)	Specificity[b] (%)	PPV[c] (%)	NPV[d] (%)
R/EX/FS	Exact	Prob-FS	90.48	99.78	97.35	99.15
R/EX/EM	Exact	Prob-EM	90.48	99.78	97.35	99.15
R/EX/EPI	Exact	Prob-EPI	90.48	99.78	97.35	99.15
MTB/EX/FS	Exact	Prob-FS	90.48	99.78	97.35	99.15
MTB/EX/EM	Exact	Prob-EM	90.48	99.78	97.35	99.15
MTB/EX/D	Exact	Det	90.48	99.78	97.35	99.15
CU/EX/FS	Exact	Prob-FS	90.48	99.78	97.35	99.15
LP/EX/FS	Exact	Prob-FS	90.48	99.78	97.35	99.15
LP/EX/EM	Exact	Prob-EM	90.48	99.78	97.35	99.15
R/INEX/FS	Inexact	Prob-FS	93.36	99.74	97.01	99.41
R/INEX/EM	Inexact	Prob-EM	90.48	99.78	97.35	99.15
MTB/INEX/FS	Inexact	Prob-FS	90.48	99.78	97.35	99.15
MTB/INEX/EM	Inexact	Prob-EM	90.48	99.78	97.35	99.15
MTB/INEX/D	Inexact	Det	90.48	99.78	97.35	99.15
CU/INEX/FS	Inexact	Prob-FS	90.51	99.78	97.33	99.16
LP/INEX/FS	Inexact	Prob-FS	0.04	100.00	86.67	91.80
LP/INEX/EM	Inexact	Prob-EM	0.04	100.00	86.67	91.80

DOB, date of birth; PPV, positive predictive value; NNV, negative predictive value; R, R package; MTB, Merge ToolBox; CU, Curtin University Probabilistic Linkage Engine; LP, Link Plus; Prob-FS, probabilistic, Fellegi-Sunter; Prob-EM, probabilistic, expectation-maximization; Prob-EPI, probabilistic, EpiLink; Det, deterministic; MRN, medical record number.

All test characteristics are based on comparison with the gold standard, inpatient MRN.

[a] Sensitivity = percentage of record pairs with matching inpatient MRNs that are declared matches.

[b] Specificity = percentage of record pairs without matching inpatient MRNs that are declared non-matches.

[c] PPV = percentage of pairs that are declared matches for which the inpatient MRNs agree.

[d] NPV = percentage of pairs declared nonmatches for which the inpatient MRNs do not agree.

Table E. Standard decision rule characteristics for the 17 DOB linkage runs when declared matches are those with the first or second highest weight.

Linkage Run Name	String Matching	Weight Determination	Sensitivity[a] (%)	Specificity[b] (%)	PPV[c] (%)	NPV[d] (%)
R/EX/FS	Exact	Prob-FS	90.53	99.78	97.34	99.16
R/EX/EM	Exact	Prob-EM	90.54	99.78	97.34	99.16
R/EX/EPI	Exact	Prob-EPI	90.54	99.78	97.34	99.16
MTB/EX/FS	Exact	Prob-FS	90.54	99.78	97.34	99.16
MTB/EX/EM	Exact	Prob-EM	90.54	99.78	97.34	99.16
MTB/EX/D	Exact	Det	98.57	99.29	92.58	99.87
CU/EX/FS	Exact	Prob-FS	90.54	99.78	97.34	99.16
LP/EX/FS	Exact	Prob-FS	90.54	99.78	97.34	99.16
LP/EX/EM	Exact	Prob-EM	90.54	99.78	97.34	99.16
R/INEX/FS	Inexact	Prob-FS	93.42	99.74	96.99	99.41
R/INEX/EM	Inexact	Prob-EM	90.49	99.78	97.35	99.16
MTB/INEX/FS	Inexact	Prob-FS	90.49	99.78	97.35	99.16
MTB/INEX/EM	Inexact	Prob-EM	90.49	99.78	97.35	99.16
MTB/INEX/D	Inexact	Det	90.49	99.78	97.35	99.16
CU/INEX/FS	Inexact	Prob-FS	90.52	99.78	97.33	99.16
LP/INEX/FS	Inexact	Prob-FS	0.05	100.00	83.33	91.80
LP/INEX/EM	Inexact	Prob-EM	0.05	100.00	83.33	91.80

DOB, date of birth; PPV, positive predictive value; NNV, negative predictive value; R, R package; MTB, Merge ToolBox; CU, Curtin University Probabilistic Linkage Engine; LP, Link Plus; Prob-FS, probabilistic, Fellegi-Sunter; Prob-EM, probabilistic, expectation-maximization; Prob-EPI, probabilistic, EpiLink; Det, deterministic; MRN, medical record number.

All test characteristics are based on comparison with the gold standard, inpatient MRN.

[a] Sensitivity = percentage of record pairs with matching inpatient MRNs that are declared matches.

[b] Specificity = percentage of record pairs without matching inpatient MRNs that are declared non-matches.

[c] PPV = percentage of pairs that are declared matches for which the inpatient MRNs agree.

[d] NPV = percentage of pairs declared nonmatches for which the inpatient MRNs do not agree.

Fig D is an expanded version of Fig 4 in the main text. Showing all values of sensitivity obscures any differences among the linkage runs.

Fig D. ROC curves for all date of birth linkage runs, for all values of sensitivity.

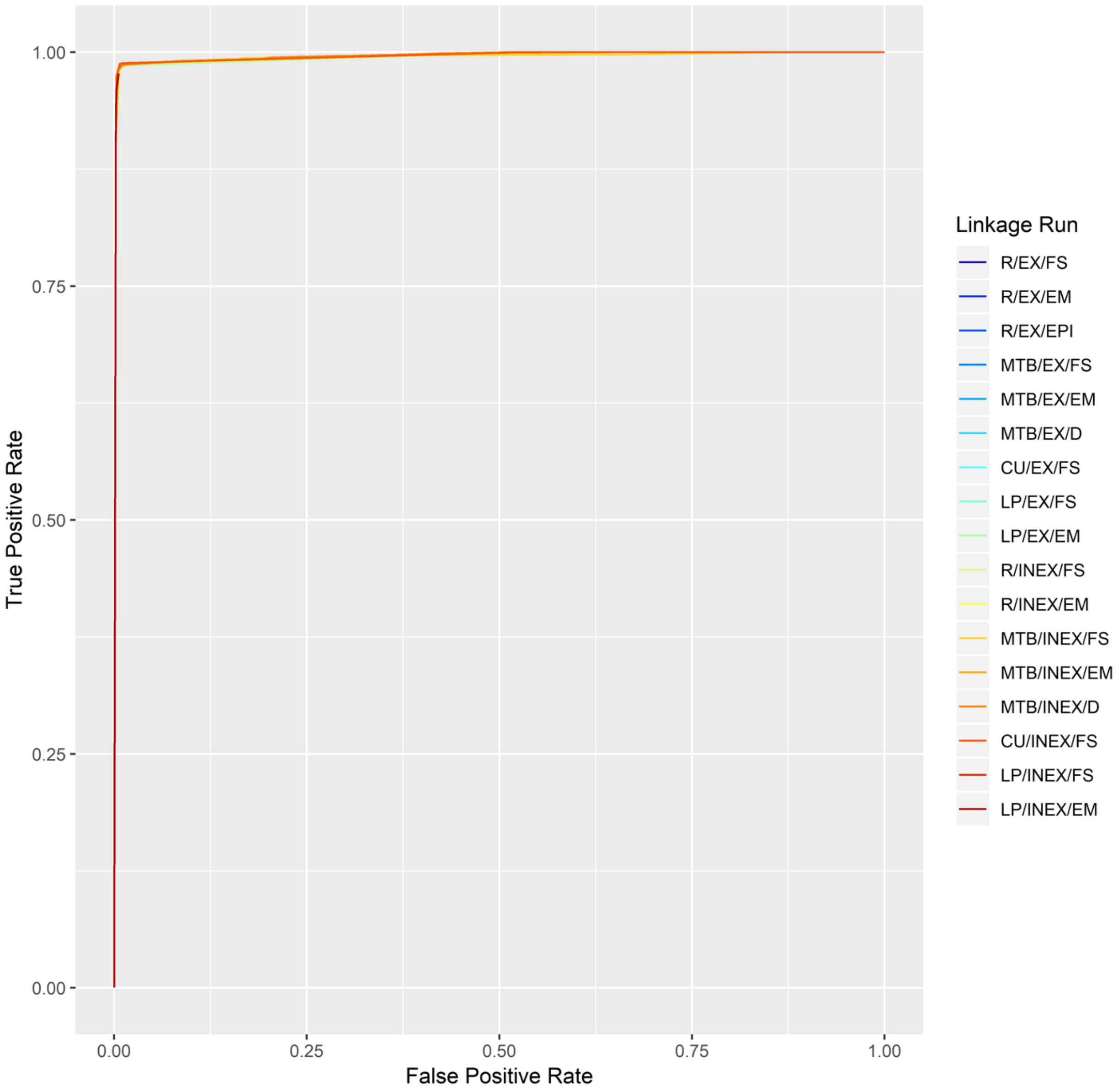

ROC, receiver operating characteristic; R, R package; MTB, Merge ToolBox; CU, Curtin University Probabilistic Linkage Engine; LP, Link Plus; EX, exact string matching; INEX, inexact string matching; FS, probabilistic, Fellegi-Sunter; EM, probabilistic, expectation-maximization; EPI, probabilistic, EpiLink; D, deterministic.

Table F contains exactly calculated values of the area under the receiver operating characteristic (ROC) curve (AUC) for 15 of the 17 values, when threshold scaled weight was the criterion for declaring a match and inpatient MRN was the gold standard. Linkage runs with inexact string matching slightly outperformed linkage runs with exact string matching, even if only at the thousandth decimal level. For example, linkage runs R/INEX/FS (0.9940) had a slightly higher AUC than R/EX/FS (0.9939), yet both used probabilistic Fellegi-Sunter weight determination. The other pairwise comparisons are R/INEX/EM and R/EX/EM (probabilistic, expectation-maximization); MTB/INEX/FS and MTB/EX/FS (probabilistic Fellegi-Sunter); MTB/INEX/EM and MTB/EX/EM (probabilistic, expectation-maximization); and MTB/INEX/D and MTB/EX/D (deterministic).

Table F. AUC for all DOB linkage runs[a].

Linkage Run Name	String Matching	Weight Determination	AUC
R/EX/FS	Exact	Prob-FS	0.9939
R/EX/EM	Exact	Prob-EM	0.9939
R/EX/EPI	Exact	Prob-EPI	0.9939
MTB/EX/FS	Exact	Prob-FS	0.9939
MTB/EX/EM	Exact	Prob-EM	0.9939
MTB/EX/D	Exact	Det	0.9938
CU/EX/FS	Exact	Prob-FS	0.9938
LP/EX/FS	Exact	Prob-FS	0.9939
LP/EX/EM	Exact	Prob-EM	0.9939
R/INEX/FS	Inexact	Prob-FS	0.9940
R/INEX/EM	Inexact	Prob-EM	0.9943
MTB/INEX/FS	Inexact	Prob-FS	0.9940
MTB/INEX/EM	Inexact	Prob-EM	0.9946
MTB/INEX/D	Inexact	Det	0.9948
CU/INEX/FS	Inexact	Prob-FS	0.9946
LP/INEX/FS	Inexact	Prob-FS	[b]
LP/INEX/EM	Inexact	Prob-EM	[b]

AUC, area under the receiver operating characteristic curve; DOB, date of birth; R, R package; MTB, Merge ToolBox; CU, Curtin University Probabilistic Linkage Engine; LP, Link Plus; Prob-FS, probabilistic, Fellegi-Sunter; Prob-EM, probabilistic, expectation-maximization; Prob-EPI, probabilistic, EpiLink; Det, deterministic; MRN, medical record number.

[a] AUC values based on use of scaled weights as the decision criterion compared to the gold standard, inpatient medical record number.

[b] AUC incalculable due to missing low-value weights in Link Plus.

Ensemble method: Rank 1 or 2 voting

Many researchers conducting linkage work use the highest or the two highest weights when declaring pairs to be matches. Consequently, for our second proposed rank ensemble method, we proposed the algorithm “Rank 1 or 2 Voting,” where the value assigned to each pair ranged from 0 to 17, computed by assessing the number of linkage runs that assigned the highest or second highest weight to that pair. The Rank 1 or 2 voting ensemble method declared as matches those pairs receiving at least k votes, where k ranges from 1 (the least conservative choice) to 17 (the most conservative choice). Fig E shows the ROC curve for this method compared to the average scaled weight ensemble method and the single matching rung with the highest AUC, deterministic linkage with inexact string matching using MBT (MBT/INEX/D).

Fig E. ROC curves for ensemble methods and best single matching method, sensitivity ≥.90.

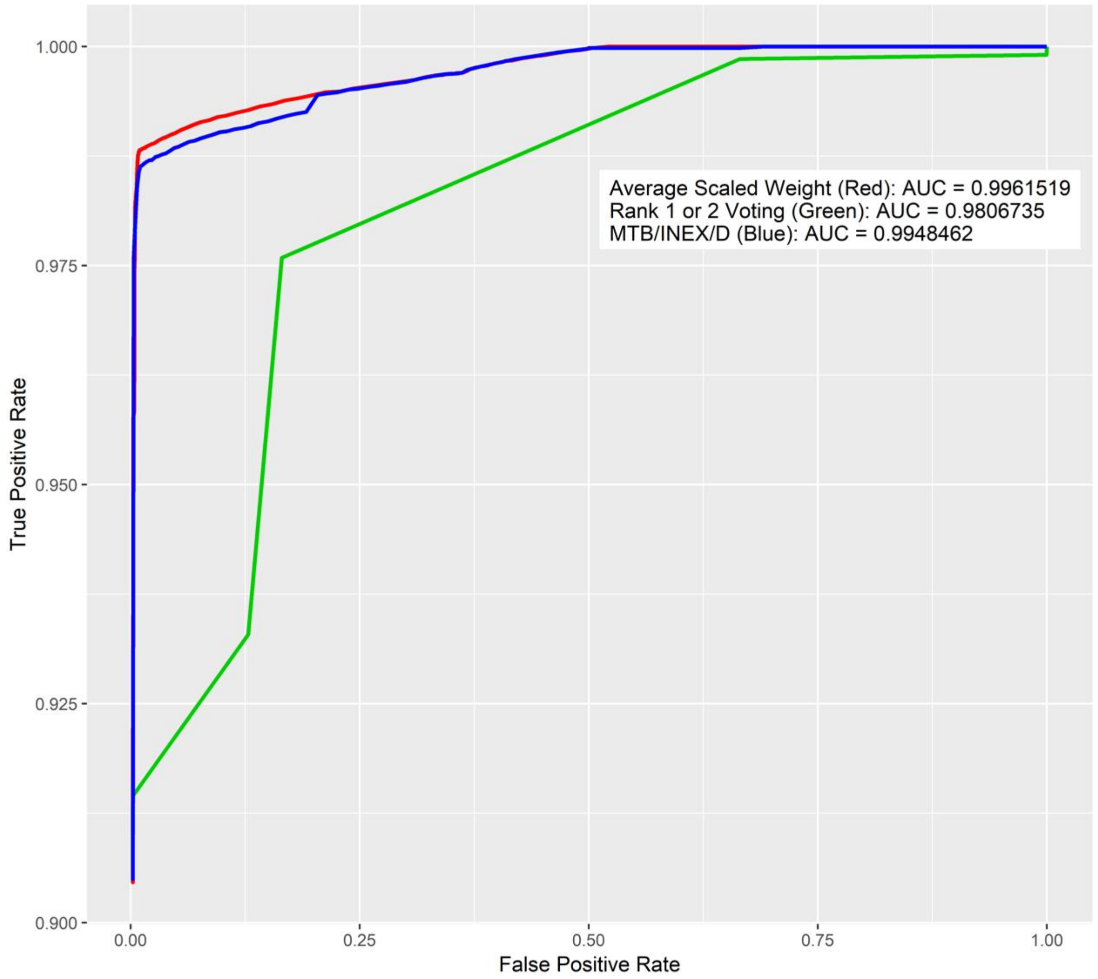

AUC, area under the receiver operating characteristic curve; ROC, receiver operating characteristic; MTB, Merge ToolBox; INEX, inexact string matching; D, deterministic.

Year of birth experiment

In the DOB experiment, if records differed on either day or month of birth, they would not be declared a match by any of the methods despite matching on all other linkage variables. Although the 3 MTB inexact string matching linkage runs also yield the same 30,805 highest rank matches, it is unclear why the CU/EX/FS produces an additional 23 matches. As was also true for blocking on full date of birth, both LP/EX/FS and LP/INEX/EM declare only a handful of matches based on highest or second highest weights. The number of matches based on second highest weight varies considerably among the linkage runs, varying from 3 to 402,743, with both Prob-EM exact string matching runs producing large and identical numbers of matching pairs with second highest weight.

Notably, blocking on YOB imposed major computational challenges because of the 329-fold increase in the number of compared pairs (131,906,591), which has implications for running time and memory, both of which are approximately proportional to the number of compared pairs.

Table G. Summary of weights produced by the 15 linkage runs using YOB as the blocking variable.

Linkage Run Name	String matching	Weight Determination	Number of Weights	Minimum Weight	Maximum Weight	Pairs with Highest Weight	Pairs with Second Highest Weight	Pairs with Lowest Weight[d]	Pairs with Second Lowest Weight[b]
R/EX/FS	Exact	Prob-FS	8	-12.3808	32.35027	30,805	26	63,469,487	67,973,584
R/EX/EM	Exact	Prob-EM	8	-15.2857	23.61731	30,805	371,180	63,469,487	28,065
R/EX/EPI	Exact	Prob-EPI	8	0	1	30,805	26	63,469,487	67,973,584
MTB/EX/FS	Exact	Prob-FS	10	-12.3808	32.35027	30,805	26	63,466,474	3,013
MTB/EX/EM	Exact	Prob-EM	10	-12.9681	23.51965	30,805	371,180	63,466474	28,065
MTB/EX/D	Exact	Det	4	0	3	30,805	402,743	63,469,487	68,003,556
CU/EX/FS	Exact	Prob-FS	10	-12.3808	32.35027	30,805	26	63,464,504	3,012
LP/EX/FS	Exact	Prob-FS	10	-7.53877	12.78385	30,805	26	63,466,474	3,013
LP/EX/EM	Exact	Prob-EM	10	-7.53877	12.78385	30,805	26	63,466,474	3,013
MTB/INEX/FS	Inexact	Prob-FS	916,806	-12.3808	32.35027	30,805	3	2,040,621	139
MTB/INEX/EM	Inexact	Prob-EM	916,807	-12.9681	23.51965	30,805	3	2,040,621	1
MTB/INEX/D	Inexact	Det	266,929	0	3	30,805	3	2,040,760	1
CU/INEX/FS	Inexact	Prob-FS	15,663	-12.3808	32.35027	30,828	3	62,405,370	2
LP/INEX/FS	Inexact	Prob-FS	45	1.9	15.8	15	3	[a]	[a]
LP/INEX/EM	Inexact	Prob-EM	45	1.9	15.8	15	3	[a]	[a]

YOB, year of birth; R, R package; MTB, Merge ToolBox; CU, Curtin University Probabilistic Linkage Engine; LP, Link Plus; Prob-FS, probabilistic, Fellegi-Sunter; Prob-EM, probabilistic, expectation-maximization; Prob-EPI, probabilistic, EpiLink; Det, deterministic.

[a] We were unable to recover negative weights for Link Plus with inexact string matching.

Table H. Agreement with gold standard among records with the highest weights, blocking on YOB.

Linkage Run Name	String matching	Weight Determination	Number (%) of Pairs with Highest Weight	
			Agreement with inpatient MRN	
			No	Yes
R/EX/FS	Exact	Prob-FS	1,035 (3.4)	29,770 (96.6)
R/EX/EM	Exact	Prob-EM	1,035 (3.4)	29,770 (96.6)
R/EX/EPI	Exact	Prob-EPI	1,035 (3.4)	29,770 (96.6)
MTB/EX/FS	Exact	Prob-FS	1,035 (3.4)	29,770 (96.6)
MTB/EX/EM	Exact	Prob-EM	1,035 (3.4)	29,770 (96.6)
MTB/EX/D	Exact	Det	1,035 (3.4)	29,770 (96.6)
CU/EX/FS	Exact	Prob-FS	1,035 (3.4)	29,770 (96.6)
LP/EX/FS	Exact	Prob-FS	1,035 (3.4)	29,770 (96.6)
LP/EX/EM	Exact	Prob-EM	1,035 (3.4)	29,770 (96.6)
MTB/INEX/FS	Inexact	Prob-FS	1,035 (3.4)	29,770 (96.6)
MTB/INEX/EM	Inexact	Prob-EM	1,035 (3.4)	29,770 (96.6)
MTB/INEX/D	Inexact	Det	1,035 (3.4)	29,770 (96.6)
CU/INEX/FS	Inexact	Prob-FS	1,047 (3.4)	29,781 (96.6)
LP/INEX/FS	Inexact	Prob-FS	2 (13.3)	13 (86.7)
LP/INEX/EM	Inexact	Prob-EM	2 (13.3)	13 (86.7)

YOB, year of birth; R, R package; MTB, Merge ToolBox; CU, Curtin University Probabilistic Linkage Engine; LP, Link Plus; Prob-FS, probabilistic, Fellegi-Sunter; Prob-EM, probabilistic, expectation-maximization; Prob-EPI, probabilistic, EpiLink; Det, deterministic.

Table I. Standard decision rule characteristics for the 15 YOB linkage runs when declared matches are those with the highest weight.

Linkage Run Name	String matching	Weight Determination	Sensitivity[a] (%)	Specificity[b] (%)	PPV[c] (%)	NPV[d] (%)
R/EX/FS	Exact	Prob-FS	90.48	100.00	96.64	100.00
R/EX/EM	Exact	Prob-EM	90.48	100.00	96.64	100.00
R/EX/EPI	Exact	Prob-EPI	90.48	100.00	96.64	100.00
MTB/EX/FS	Exact	Prob-FS	90.48	100.00	96.64	100.00
MTB/EX/EM	Exact	Prob-EM	90.48	100.00	96.64	100.00
MTB/EX/D	Exact	Det	90.48	100.00	96.64	100.00
CU/EX/FS	Exact	Prob-FS	90.48	100.00	96.64	100.00
LP/EX/FS	Exact	Prob-FS	90.48	100.00	96.64	100.00
LP/EX/EM	Exact	Prob-EM	90.48	100.00	96.64	100.00
MTB/INEX/FS	Inexact	Prob-FS	90.48	100.00	96.64	100.00
MTB/INEX/EM	Inexact	Prob-EM	90.48	100.00	96.64	100.00
MTB/INEX/D	Inexact	Det	90.48	100.00	96.64	100.00
CU/INEX/FS	Inexact	Prob-FS	90.51	100.00	96.60	100.00
LP/INEX/FS	Inexact	Prob-FS	0.04	100.00	86.67	99.98
LP/INEX/EM	Inexact	Prob-EM	0.04	100.00	86.67	99.98

YOB, year of birth; PPV, positive predictive value; NNV, negative predictive value; R, R package; MTB, Merge ToolBox; CU, Curtin University Probabilistic Linkage Engine; LP, Link Plus; Prob-FS, probabilistic, Fellegi-Sunter; Prob-EM, probabilistic, expectation-maximization; Prob-EPI, probabilistic, EpiLink; Det, deterministic.

All test characteristics are based on comparison with the gold standard, inpatient MRN.

[a] Sensitivity = percentage of record pairs with matching inpatient MRNs that are declared matches.

[b] Specificity = percentage of record pairs without matching inpatient MRNs that are declared non-matches.

[c] PPV = percentage of pairs that are declared matches for which the inpatient MRNs agree.

[d] NPV = percentage of pairs declared nonmatches for which the inpatient MRNs do not agree.

Table J. Computation time for date of birth linkage runs.

Linkage Run Name	String Matching	Weight Determination	Computational Time (seconds)[a]
R/EX/FS	Exact	Prob-FS	14
R/EX/EM	Exact	Prob-EM	14
R/EX/EPI	Exact	Prob-EPI	7
MTB/EX/FS	Exact	Prob-FS	16
MTB/EX/EM	Exact	Prob-EM	7
MTB/EX/D	Exact	Det	8
CU/EX/FS	Exact	Prob-FS	~5
LP/EX/FS	Exact	Prob-FS	~55
LP/EX/EM	Exact	Prob-EM	~55
R/INEX/FS	Inexact	Prob-FS	10
R/INEX/EM	Inexact	Prob-EM	12
MTB/INEX/FS	Inexact	Prob-FS	10
MTB/INEX/EM	Inexact	Prob-EM	9
MTB/INEX/D	Inexact	Det	11
CU/INEX/FS	Inexact	Prob-FS	~5
LP/INEX/FS	Inexact	Prob-FS	~55
LP/INEX/EM	Inexact	Prob-EM	~55

R, R package; MTB, Merge ToolBox; CU, Curtin University Probabilistic Linkage Engine; LP, Link Plus; Prob-FS, probabilistic, Fellegi-Sunter; Prob-EM, probabilistic, expectation-maximization; Prob-EPI, probabilistic, EpiLink; Det, deterministic.
[a] ~ indicates timing not provided by linkage program.

Table K. Computational time for year of birth linkage runs.

Linkage Run Name	String Matching	Weight Determination	Computational Time (seconds)[a]
R/EX/FS	Exact	Prob-FS	3,825
R/EX/EM	Exact	Prob-EM	3,352
R/EX/EPI	Exact	Prob-EPI	3,020
MTB/EX/FS	Exact	Prob-FS	879
MTB/EX/EM	Exact	Prob-EM	1,283
MTB/EX/D	Exact	Det	1,645
CU/EX/FS	Exact	Prob-FS	375
LP/EX/FS	Exact	Prob-FS	70
LP/EX/EM	Exact	Prob-EM	66
MTB/INEX/FS	Inexact	Prob-FS	915
MTB/INEX/EM	Inexact	Prob-EM	1,101
MTB/INEX/D	Inexact	Det	1630
CU/INEX/FS	Inexact	Prob-FS	375
LP/INEX/FS	Inexact	Prob-FS	71
LP/INEX/EM	Inexact	Prob-EM	71

R, R package; MTB, Merge ToolBox; CU, Curtin University Probabilistic Linkage Engine; LP, Link Plus; Prob-FS, probabilistic, Fellegi-Sunter; Prob-EM, probabilistic, expectation-maximization; Prob-EPI, probabilistic, EpiLink; Det, deterministic.

Supporting information references

1. Borg A, Sariyar M. Package 'RecordLinkage' 2016 [updated July 27; cited 2018 August 16]. Available from: https://cran.r-project.org/web/packages/RecordLinkage/RecordLinkage.pdf.
2. Centers for Disease Control and Prevention. [To Parent Directory] Atlanta, GA: Centers for Disease Control and Prevention; 1999 [cited 2018 October 8]. Available from: ftp://ftp.cdc.gov/pub/Software/.
